# The Institutional Library

**Published:** 1914-06-06

**Authors:** 


### THE INSTITUTIONAL LIBRARY.

### A Practical Public Health Manual.

The Bacteriological Examination of Food and Water. By Wm. G. Savage, M.D., D.P.H., B.Sc. Cambridge Public Health Series. (Cambridge: At the University Press. 1914. Pp. 173. Price 7s. 6d. net.)

Under the description of public health topics a great variety of matters are included, some of which are but remotely concerned with the welfare of the community. But it may safely be said that all the volumes of the Cambridge Public Health Series now in active preparation deal or will deal with subjects of the first importance to the public health. The books included in the series present in a useful and handy form the knowledge now available in the many branches of the subject. They are written by experts, men who are continually occupied in working at investigations connected with their particular themes and their practical application. The highly technical nature of laboratory work, and the very lengthy procedures which are now necessary for the elucidation of the causes, for instance, of food poisoning or water contamination render it impossible for any except those who can work uninterruptedly in one small area of the field to glean reliable data. Such a series of volumes as is represented by the one now under notice should not fail to appeal to a much wider circle of readers than the bacteriologists and the laboratory workers. To medical officers of health several of the volumes will make an obvious appeal, but municipal engineers and architects, factory surgeons, and sanitary inspectors and school medical officers will also find books dealing with matters concerning their work very intimately. Many of the volumes will contain material which will be suggestive and instructive to members of hygiene and public health committees, and it is intended that they shall influence the large body of educated and intelligent public opinion which is now becoming increasingly interested in the problems of public health.

Dr. Savage, who is the county medical officer for Somerset, has contributed a volume on a highly important but very technical branch of public health laboratory work. It is true that most text-books on bacteriology devote space to the examination of water, air, and foods, but this is generally quite inadequate and uselessly incomplete; the text-books are naturally more concerned with the examination of morbid human material. Dr. Savage's manual is of a particularly practical character, and deals with the examination of water, sewage, shell-fish, milk, meat, and air always from the point of view of what may be called sanitary bacteriology, which is, after all, only another way of saying that the subjects are considered from the standpoint of sanity and common sense.

### A Lady's Attempt at a Text-book.

Keep Breathing. By Mme. M. A. Carlisle Carr. (London: Elliot Stock. 1914. Pp. 54. Price 2s. net.)

We should be the last to deny the value of breathing exercises in certain cases, or the importance of correct respiratory methods. But we really cannot see the advantage of the course which "Madame" Carlisle Carr has taken in composing this "text-book" of instruction in what she conceives to be correct methods of breathing. It consists of a perpetual string of questions and answers, on the "Child's Guide to Knowledge" principle. The questions are apparently those of someone whose mind is perfectly blank; but, curiously enough, they are all designed to elicit the dogmatic assertions of the imaginary teacher. These assertions are in some cases palpably untrue, and in others are mere *obiter dicta* incapable of proof or disproof: many of them are essentially question-begging into the bargain. The author may have the very best intentions, but she is not intellectually equipped for the production of text-books. Incidentally we object to her assumption of the title of "Professor," unless she can show that some acknowledged educational authority has appointed her to a chair.

Case Book for Massage and Weir-Mitchell Treatment. Specially ruled to meet the requirements of the I.S.T.M. (The Scientific Press. Pp. 56. Price 6d.)

This well designed book of case-charts can be strongly recommended to those who carry out either massage or Weir-Mitchell treatment or both. It will save them much time and labour in recording their cases, and fills a very decided want. Our only complaint is that Weir is spelt Wier on the cover, though it is correctly shown inside.

### The Cult of Tuberculosis.

The Early Diagnosis of Tubercle. By Clive Riviere, M.D., M.R.C.P. (London: Henry Frowde and Hodder and Stoughton. 1914. Oxford Medical Publications. Pp. 270. Price 6s. net.)

The obvious thing to say about this book is that it is opportune. The early diagnosis of pulmonary tuberculosis has, at the present day, attained almost the dimensions of a cult; in fact, it is not too much to declare that among a few tuberculosis "experts" it has become little less than an obsession. Dr. Clive Riviere has done well to gather into a compact volume all that need be said, for the present at any rate, on the early diagnosis of tubercle of the lungs. The book is concerned entirely with thoracic tuberculosis and is conveniently divided into two parts: adults and children. The latter deals with the disease in the mediastinal glands and at the hilus. The diagnosis of this disease has now become a very complex affair; the author has obviously tried hard to emphasise the really reliable bits of evidence to be gleaned from a careful examination of the chest, and has, it will be admitted, compiled what is the most useful little book on the subject that has so far appeared. On the special methods of diagnosis the author has written a helpful and practical section and rightly accords to tuberculin tests a subsidiary position. The *x*-rays and the sputum are both discussed thoroughly and the technical details fully expounded. The quantitative cutaneous test is described in elaborate detail.

A great many readers will be grateful for the second portion of the book—that dealing with thoracic disease in children. This subject is a continual source of difficulty to school medical officers, and even among tuberculosis workers there is hardly any agreement as to what is tubercular and what is not. The lack of a direct lead from his own experience is the chief fault in this section, which is otherwise a good summary of the special points and an excellent guide to the examination of children. It is unfortunate that Dr. Riviere does not draw sufficient attention to those not rare cases of fibrosis of the lung with displacement of the heart in children who are not tubercular, nor to those cases in which râles persist for months or even years after pneumonia. Presumably by an oversight, fulness of veins and enlarged glands are included under symptoms. Dr. Riviere has devoted careful pages to the highly important recognition of enlargement of the tracheo-bronchial glands. The care of the child is surely the root problem in dealing with tuberculosis, and the diagnosis of latent tubercular infection of deep-seated glands is a subject to which studious attention will have to be paid. It is quite certain that this volume will command a ready sale, and that before long another edition will be called for, when the author, it may be hoped, will be able to embody the suggestions referred to above.

### The Common Sense of Psycho-Analysis.

Psychopathology of Everyday Life. By Professor Sigmund Freud, LL.D. Authorised English Edition, with an Introduction by A. A. Brill, Ph.B., M.D. (T. Fisher Unwin. 12s. 6d. net.)

This book is a symptom of a welcome tendency in the study of psychopathology and provides, from one point of view, a very desirable reaction from the methods of Lombroso, Kraft-Ebing, and similar writers. These writers, being specialists, had all the faults of specialism as well as its virtues. They were painstaking in the collection of facts, but the deductions which they drew from them led to the result that, quite excusably, every reader found in himself the direst symptoms whenever he suffered from any over-fatigue, excitement, or even indulged the whims of high spirits by seeking relief in nonsense rhymes, castles in the air, or any of the imaginative delights of exuberant health or levity. They coined a series of appalling terms to describe the simplest states of mind. If one felt depressed, one was a mattoid; if one felt self-confident, one was a megalomaniac; if one did not like to cross a street, one was suffering from agoraphobia; if one was facile of expression, one was a graphomaniac; and so forth. This meant that an entirely false, academic, empiric standard of normal health was set up, and as every human being showed the most startling differences from it, we were all lumped as degenerates, if not worse. Professor Freud at once gains our respect by carefully avoiding such pretentious dogmatism, and by going direct to the psychopathology of *everyday life*, and considering it scientifically, without calling it names; he shows that such processes as that of forgetting proper names or foreign words to which everyone is liable are explicable, not on the absurd ground that they are grave symptoms of mental or psychic disturbance, but by certain laws of memory which he works out with delightful acumen and precision. It is the scientific method, popularised by Dr. Conan Doyle in Sherlock Holmes, of observation and deduction. As he has no brief to convict humanity of madness or degeneration, but a scientific curiosity to learn the facts and arrive at the explanation of the laws of their working, his eyes are not blinded as Lombroso's, for instance, quite frequently were. In the case of the forgetting of proper names he arrives at the conclusion that "besides the simple forgetting . . . there is another forgetting which is motivated by repression." One train of thought is interrupted by another, and in the disturbance consequent upon it an incorrect substitute for the desired name is suggested. What the substitute is depends, as he shows by very brilliant examples which reveal a genuine "detective" faculty of observation and deduction, upon the latent train of ideas—hopes, fears, worries, preoccupations—in the forgetter's mind. "A second mechanism of forgetting," he tells us, "is the disturbance of thought through an inner contradiction emanating from repression." Mistakes in speech and in reading and writing provide material for other chapters on the same lines, and symptomatic and chance actions, errors, and combined faulty acts are also discussed. But, finally, we cannot be too thankful that the two poles of psychopathology and everyday life have been brought together, for by this happy conjunction the science is rescued from the abyss of nescience into which the devotion of investigators almost entirely to abnormal types was leading it, and everyday life is now found equally fruitful to the student. For because it is everyday life the science is rescued from that worst kind of specialism which, because it deals with "idiots" (isolated examples), attempts to its own confusion to import their vagaries into states of health. This has converted all men into monsters by setting up a false standard of health, and has overlooked even in monsters those elements of common humanity which even a diseased human being possesses in his degree with healthy types. For rescuing us from this Professor Freud deserves the high praise and true gratitude of genuine scientists and all who have had the glass held up to them only to be shown Caliban's face in place of their own there. The price is excessive, and the paper too thick.

A System of Surgery. Edited by C. C. Choyce, M.B., F.R.C.S. Vol. III. (Cassell and Co. 1914. Pp. 901. Price 21s. net.)

The concluding volume of this system deals with the surgery of the cardio-vascular system—nose, ear, larynx, lungs, nerves, brain, bones, muscles, and joints. It fully maintains throughout the volume the reputation already won by the preceding ones. It is not very profitable to discriminate between the sections of a work which maintains so high a general level; but a word of especial praise is due to the lucidity with which the subject of cut throat has been handled in an exceedingly brief space. The arrangement of the sections, the selection of types for differentiating them, and the whole of the "get up" of the book reflect great credit upon both the editor and the publishers. There are, of course, points in the discussions of the various diseases upon which the teaching here expressed will not command unanimous assent. But in the main it can be said unreservedly that this book reflects the best standards of British surgery. Last, but not least, mention should be made of a splendid index.

### A Pocket Guide to Ionic Medication.

The Theory and Practice of Ionic Medication. By Lytton Maitland, M.D. (The Scientific Press. Pocket Guide Series. Pp. 102. Price 1s. net.)

Exactly what position ionic medication will hold in ten years' time he would be a rash man that would predict now. It may have ousted a large proportion of the older therapeutic methods, or it may be forgotten. But in the present year of grace this much is certain: that ionic medication has scored some remarkable successes, and that it well deserves a fuller trial from the medical profession than it has had as yet. Apart from those who specialise in electro-therapeutics, the proportion of medical men who know anything about ionisation is small. There is no longer any excuse for this condition of ignorance. Dr. Maitland has provided a pocket guide to the subject which can be read and assimilated in an hour or so, not only by an expert, but by any general practitioner or well-trained nurse. We do not, of course, imply that anyone can become an accomplished ioniser merely by reading this booklet; but at least anyone can understand sufficient of the subject to take it up and apply the methods here described. From the general practitioner's point of view the brevity and condensation necessary to present the principles of ionic medication in so small a compass are a great advantage.

